# PINK1 Deficiency Ameliorates Cisplatin-Induced Acute Kidney Injury in Rats

**DOI:** 10.3389/fphys.2019.01225

**Published:** 2019-09-25

**Authors:** Li Zhou, Ling Zhang, Yu Zhang, Xuan Yu, Xiuping Sun, Tao Zhu, Xianglei Li, Wei Liang, Yunlin Han, Chuan Qin

**Affiliations:** ^1^Key Laboratory of Human Disease Comparative Medicine, Chinese Ministry of Health, Institute of Laboratory Animal Science, Chinese Academy of Medical Sciences (CAMS), Beijing, China; ^2^Key Laboratory of Human Diseases Animal Models, State Administration of Traditional Chinese Medicine, Peking Union Medicine College (PUMC), Beijing, China

**Keywords:** PINK1, nephrotoxicity, mitophagy, DRP1, mitochondrial dynamics

## Abstract

Mitophagy plays a key role in cleaning damaged and depolarized mitochondria to maintain cellular homeostasis and viability. Although it was originally found in neurodegenerative diseases, mitophagy is reported to play an important role in acute kidney injury. PINK1 and Parkin are key molecules in mitophagy pathway. Here, we used PINK1 knockout rats to examine the role of PINK1/Parkin-mediated mitophagy in cisplatin nephrotoxicity. After cisplatin treatment, PINK1 knockout rats showed lower plasma creatinine and less tubular damage when compared with wild-type rats. Meanwhile, mitophagy indicated by autophagosome formation and LC3B-II accumulation was also attenuated in PINK1 knockout rats. Renal expression of PINK1 and Parkin were down-regulated while BNIP3L was up-regulated by cisplatin treatment, indicating a major role of BNIP3/BNIP3L pathway in cisplatin-induced mitophagy. Transmission electron microscopy showed that PINK1 deficiency inhibited cisplatin-induced mitochondrial fragmentation indicating an involvement of mitochondrial fusion and fission. Renal expression of mitochondrial dynamics related proteins including *Fis1*, *Drp1*, *Mfn1*, *Mfn2*, and *Opa1* were checked by real-time PCR and western blots. The results showed PINK1 deficiency distinctly prevented cisplatin-induced up-regulation of DRP1. Finally, PINK1 deficiency alleviated cisplatin-induced tubular apoptosis indicated by TUNEL assay as well as the expression of caspase3 and cleaved caspase3. Together, these results suggested PINK1 deficiency ameliorated cisplatin-induced acute kidney injury in rats, possibly via inhibiting DRP1-mediated mitochondrial fission and excessive mitophagy.

## Introduction

Cisplatin is a widely used chemotherapy drug. It is highly effective against numerous cancers including bladder, testicular, ovarian, head and neck, uterine cervical carcinoma, non-small cell lung carcinoma and so forth. However, the use of cisplatin is limited by numerous undesirable side effects in normal tissues. Nephrotoxicity is a common side effect that occurs in up to one-third of patients receiving cisplatin therapy (Dilruba and Kalayda, 2016). Despite extensive study, the mechanism underlying cisplatin-induced nephrotoxicity remains unclear.

Mitochondrial damage and dysfunction is closely related to the pathogenesis of acute kidney injury (AKI), especially for the damage and death of renal tubular cells (Ishimoto and Inagi, 2016). Mitochondrial injury can cause a series of cell dysfunction including oxidative stress, inflammation, and cell death (Che et al., 2014). The researches in the past decade have revealed that mitochondrial dysfunction may play a central pathogenic role in cisplatin nephrotoxicity (Yang et al., 2014). In an aqueous environment, cisplatin is subject to nucleophilic displacement of chloride forming aquated species to generate a positively charged electrophile. This electrophile preferentially accumulates within the negatively charged mitochondria. Due to less efficient DNA repair mechanisms, mitochondrial DNA is more susceptible to cisplatin than nuclear DNA. Studies have shown that the cellular sensitivity to cisplatin-induced cell death appears to correlate with both the mitochondrial membrane potential and the density of mitochondria (Miller et al., 2010). Our previous study also showed that improving mitochondrial function could ameliorate cisplatin-induced nephrotoxicity in mice (Zhou et al., 2017).

Mitophagy is a fundamental process of purging damaged or excessive mitochondria to maintain intracellular homeostasis (Kaushal and Shah, 2016). Up-regulation of autophagy or mitophagy in the kidney has been observed in several animal experimental models including ischemia-reperfusion injury (Decuypere et al., 2015; Tang et al., 2018), cisplatin nephropathy (Zhao et al., 2017a, b; Ichinomiya et al., 2018; Wang et al., 2018), cyclosporine nephropathy (Pallet et al., 2008) and protein overload (Hartleben et al., 2010).

A large number of studies have shown that autophagy is a double-edged sword. It may play a pro-survival or a pro-death role (Shintani and Klionsky, 2004). In most studies, autophagy plays a protective role in kidney injury. Autophagy suppression by the PtdIns3K inhibitor 3-methyladenine (3-MA) or proximal tubule specific Atg5 or Atg7 KO and the lysosomal inhibitor chloroquine aggravated kidney damage in renal ischemia-reperfusion mice models (Jiang et al., 2010, 2012; Kimura et al., 2011; Liu et al., 2012). However, despite abundant evidences of the protective properties, autophagy has also been shown to be detrimental. In uninephrectomized rats, adenoviral vector carrying Bcl-2 and Bcl-XL protected renal ischemia-reperfusion injury by both the anti-apoptotic and anti-autophagic mechanisms (Chien et al., 2007). Besides, the mTOR inhibitor everolimus stimulated autophagy in rat kidney and aggravated renal ischemia-reperfusion injury (Chien et al., 2007). Moreover, ischemic and repetitive hypoxic preconditioning was reported to have protective effects in renal ischemia rats, accompanied by a decrease of autophagy (Wu et al., 2009; Yeh et al., 2010). The role of autophagy and mitophagy in cisplatin-induced kidney injury is still not fully understood.

In mammalian cells, the PINK1/Parkin pathway is considered the main pathway of mitophagy under cell stress. When mitochondria are healthy, PINK1 is imported to the inner mitochondrial membrane to be cleaved and degraded. In damaged mitochondria, the import of PINK1 into mitochondria is suppressed, leading to the accumulation of PINK1 on mitochondrial outer membrane where it recruits cytosolic Parkin. Then Parkin is activated and ubiquitinates a series of mitochondrial outer membrane proteins. These ubiquitin-tagged proteins are then recognized as a signal for the sequestration and degradation of damaged mitochondria (McWilliams and Muqit, 2017).

Here we used PINK1 knockout (KO) rats and found that PINK1 deficiency ameliorated cisplatin-induced acute kidney injury. Surprisingly, the results were contrary to the protective role of PINK1 that previous observed in mice models (Wang et al., 2018). This study may indicate a bidirectional role of PINK1 as well as a probable different mechanism between rats and mice in cisplatin nephrotoxicity.

## Materials and Methods

### Animals and Models of Cisplatin Injury

PINK1 KO and WT rats on Sprague Dawley background were obtained from Institute of Laboratory Animal Science, Peking Union Medical College and Chinese Academy of Medical Sciences, and these rat lines were previously described (Dave et al., 2014). Quantitative real-time PCR analysis of *Pink1* expression from WT and KO rats was determined using the Power Up SYBR Green master mix (Thermo Fisher Scientific) with *Pink1* expression normalized to *Actb*. Male rats of 24–28 weeks age were used in this study. The rats were housed in cages at temperature 22 ± 2°C, humidity 40 ± 5%, under a 12-hour light/dark cycle, and received sterilized maintenance diet (Beijing HFK Bioscience Co. Ltd., cat. no. 1022) and water *ad libitum*. All animal experiments were approved by the Institutional Animal Care and Use Committee of the Institute of Laboratory Animal Science of Peking Union Medical College (ILAS-QC19001). The animals were divided into four groups: WT rats control group (WT + saline; *n* = 4), KO rats control group (KO + saline; *n* = 4), WT rats treated with cisplatin group (WT + cisplatin; *n* = 6), and KO rats treated with cisplatin group (KO + cisplatin; *n* = 6). Cisplatin (Sigma-Aldrich Co. LLC., cat. no. P4394) was freshly prepared in saline at 1 mg/ml just before use. Cisplatin group rats received a single intraperitoneal injection of cisplatin at a dose of 5 mg/kg body weight, and control group rats received the equal volume of saline (Goulding and Johns, 2015). All rats were sacrificed at 96 h after cisplatin injection. Blood samples were collected from inferior vena cava for plasma creatinine and blood urea nitrogen (BUN) measurement, and renal tissues were obtained for histological and immunoblot analysis.

### Creatinine and BUN

After 96 h cisplatin treatment, all rats were anesthetized with 3% pentobarbital sodium (50mg/kg), 4 ml blood samples were collected from the inferior vena cave and anticoagulated with EDTA. The plasma was isolated by centrifugation at 4000 rpm for 10 min at 4°C. The plasma creatinine and BUN were analyzed using creatinine assay kit (Leagene Biotechnology Co. Ltd., Beijing, China) and urea assay kit (Biosino Bio-Technology and Science Inc., Beijing, China) according to the instruction of the manufactures.

### Histopathological Examination

Rat kidneys were immersion-fixed in 4% paraformaldehyde solution (PFA) overnight, and then dehydrated and embedded in paraffin. 4 μm thick sections were prepared and stained with periodic acid schiff reaction (PAS) using routine method. Renal tubular injury was indicated by tubular epithelial cell flattening, brush border loss, cytoplasmic vacuolization, cell necrosis, sloughing of cells into tubular lumen and formation of tubular casts. The degree of tissue damage was scored based on the percentage of damaged tubules by a pathologist as previously described: 0, no damage; 1, < 25%; 2, 25–50%; 3, 50–75%; 4, > 75% (Jia et al., 2011; Liu et al., 2013).

### Immunofluorescence Staining

The tissues were fixed in 4% PFA for 24 h and then embedded in paraffin. After dewaxing and rehydration, heat-mediated epitope retrieval was performed by microwave pretreatment in citrate buffer (pH 6.0). The slides were blocked in goat serum for 1 h and then incubated overnight at 4°C with anti-LC3B (Cell Signaling Technology, cat. no. 2775). After washing off the primary antibody, sections were incubated for 1 h at room temperature with Alexa Fluor 594-conjugated AffiniPure Goat Anti-Rabbit IgG (H + L) (Life Technologies, Grand Island, NY, United States). Finally, the slides were counterstained with DAPI (Sigma-Aldrich, D9542). Immunofluorescence images were obtained using a laser confocal microscope (TCS LSI, Leica, Mannheim, Germany).

### Transmission Electron Microscopy

Kidney tissues were prepared for transmission electron microscopy as previously described (Gu et al., 2018). Briefly, Fresh renal cortex was fixed in 3% glutaraldehyde solution (buffered pH 7.4) overnight at 4°C, washed with 0.1 M PBS for three times, and then post-fixed in 1% osmium tetroxide for another 2 h at 4°C. After several washes with PBS, the specimens were dehydrated and embedded in Epon 812. Ultra-thin sections were cut with a diamond knife, stained with uranium acetate and lead citrate and examined by transmission electron microscope. The lengths and widths of individual mitochondrion in proximal tubular cells were measured by Image-Pro Plus 6.0 software. Approximately 50 mitochondria in a representative area of one cell and totally 200 mitochondria of each group were measured. The ratios of mitochondria lengths to widths were calculated for statistical analysis.

### Real-Time PCR

Total RNA was extracted from renal cortex tissues using Trizol following the manufacturer’s instructions. 1 μg of total RNA was denatured at 65°C for 5 min, and subjected to reverse transcription reaction using the oligo(dT)_20_ primer and SuperScript^TM^ III Reverse Transcriptase (Invitrogen^TM^) at 50°C for 1 h. Real-time PCR was performed by the Prism 7500 Real-Time PCR Detection System (Applied Biosystems) with SYBR Green Master Mix (Thermo Fisher Scientific). The reaction conditions were 95°C for 10 min, followed by 40 repeats of 95°C for 15 s and 60°C for 1 min. The sequence of oligonucleotides used in the study are listed as follows: *Actb* sense: 5′-GG CATCCTGACCCTGAAGTA-3′ and antisense: 5′-GGGGTGT TGAAGGTCTCAAA-3′; *Pink1* sense: 5′-CATGGCTTTGGATG GAGAGT-3′ and antisense: 5′-TGGGAGTTTGCTCTTCAA GG-3′; *Parkin* sense: 5′-CTGGCAGTCATTCTGGACAC-3′ and antisense: 5′-CTCTCCACTCATCCGGTTTG-3′; *Drp1* sense: 5′-ACTGGCCCCCGTCCAGCTTA-3′ and antisense: 5′-TGAT CCACATCTGCTGGAAGGT-3′; *Fis1* sense: 5′-GCACGCA GTTTGAATACGCC-3′ and antisense: 5′-CTGCTCCTCTTT GCTACCTTTGG-3′; *Mfn1* sense: 5′-TGGGGAGGTGCTGTC TCGGA-3′ and antisense: 5′-ACCAATCCCGCTGGGGA GGA-3′; *Mfn2* sense: 5′-CTGCCAACCCCAGCATGCCA-3′ and antisense: 5′-GGCGCTTGAAGGCCCTCTCC-3′; *Opa1* sense: 5′-ATCATCTGCCACGGGT TGTT-3′ and antisense: 5′-GAGAGCGCGTCAT CATCTCA-3′.

### Western Blotting Assay

The renal cortex was homogenized with RIPA Lysis Buffer. After centrifugation, protein concentration was determined by bicinchoninic acid (BCA) protein assay kit. The protein samples were then denatured in boiling water for 10 min. Equal amounts of proteins were resolved by sodium dodecyl sulfate (SDS)-polyacrylamide gel electrophoresis and transferred onto nitrocellulose membranes. The membranes were blocked with 3% bovine serum albumin in tris-buffered saline (TBS) for 1 h and incubated overnight at 4°C with following antibodies: Parkin (Cell Signaling Technology, cat. no. 2132), DRP1 (Abcam, cat. no. ab184247), LC3B (Cell Signaling Technology, cat. no. 2775), BNIP3L/Nix (Cell Signaling Technology, cat. no. 12396), caspase3 (Cell Signaling Technology, cat. no. 9662), cleaved caspase3 (Cell Signaling Technology, cat. no. 9661), and β-actin (Sigma, cat. no. A1978). Then, the membranes were incubated with horseradish peroxidase-conjugated secondary antibodies, followed by enhanced chemiluminescence reaction. Quantification was performed by the Gel and Graph Digitizing System. The full western blot bands of LC3B, Parkin, BNIP3L, DRP1, caspase3, cleaved caspase3 were shown in Supplementary Figures S1–S6.

### Analysis of Apoptosis

Apoptosis was determined by TUNEL assay by using the *In Situ* Cell Death Detection Kit (Roche, Basel, Switzerland) in rat kidney sections. Briefly, following deparaffinization and rehydration, the sections were pretreated with 0.1 M sodium citrate (pH 6.0) at 65°C for 30 min. After washing, sections were blocked with bovine serum albumin and then incubated with TUNEL reaction mixture for 1 h at 37°C in a moist chamber. DAPI was used to label the nuclei. Sections were observed with a Laser Scanning Confocal Microscope (TCS LSI, Leica, Mannheim, Germany). The numbers of the TUNEL-positive cells in the five fields of renal cortex region for each section were counted at ×40 magnification of objective, and the average was calculated. Besides, to evaluate apoptosis, the expression of caspase3 and cleaved caspase3 was also assessed by immunoblot analysis.

### Statistical Analysis

All data are presented as means ± SE. For BUN, plasma creatinine and pathology score analyses, statistical analysis was performed with two-way ANOVA followed by Tukey test for multiple comparisons. For other experiments, statistical analysis was performed with the two-tailed student’s *t*-test. A probability of less than 0.05 was considered to be statistically significant.

## Results

### Cisplatin-Induced Kidney Injury Was Ameliorated in PINK1 KO Rats

**FIGURE 1:**
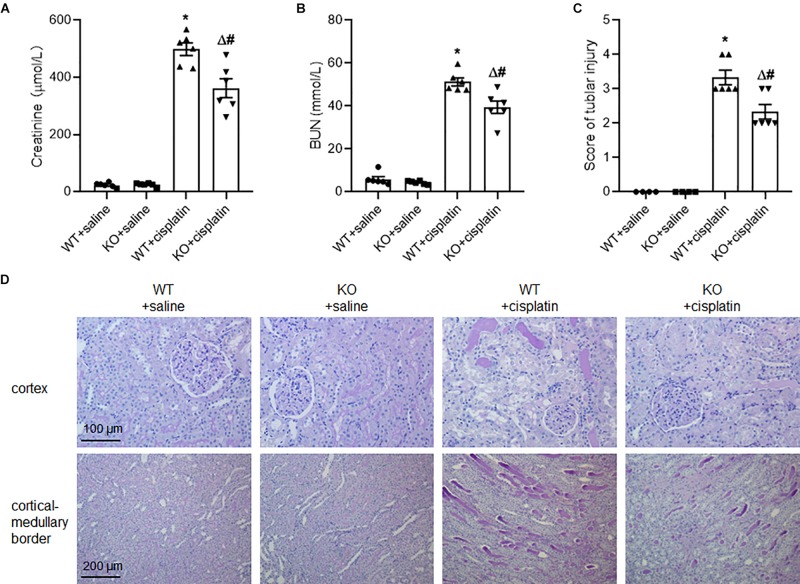
PINK1 deficiency attenuated cisplatin-induced acute kidney injury. PINK1 KO and WT rats were injected with 5 mg/kg cisplatin or saline for 72 h. **(A)** Plasma level of BUN in PINK1 WT and KO rats after cisplatin treatment. **(B)** Plasma level of creatinine in PINK1 WT and KO rats after cisplatin treatment. **(C)** Pathological score of tubular damage. **(D)** Representative histology of kidney cortex by PAS staining. *N* = 6 in each group. ^∗^*P* < 0.05, WT + cisplatin vs. WT + saline; ^#^*P* < 0.05, KO + cisplatin vs. WT + cisplatin; ^Δ^*P* < 0.05, KO + cisplatin vs. KO + saline. Data are mean ± SE.

Under unchallenged conditions, BUN and plasma creatinine were at low levels in both PINK1 KO rats and WT rats, indicating that PINK1 deficiency did not affect renal function in normal state. However, BUN and plasma creatinine levels were significantly lower in PINK1 KO rats than in WT rats at 96 h after cisplatin injection (Figures 1A,B). Next, we assessed the tubular injury via PAS staining. Histologically, the structure of renal tissues in the WT + saline group and KO + saline group was normal. Following the cisplatin treatment, WT rats displayed severe pathological changes, characterized by the distortion of the overall renal morphology, severe tubular necrosis, brush border loss and appearance of protein casts. In contrast, the cisplatin-induced damage was significantly ameliorated in PINK1 KO rats (Figures 1C,D). These results indicated that PINK1 KO rats were resistant to cisplatin-induced acute kidney injury.

### PINK1 Deficiency Prevented Cisplatin-Induced Mitophagy in Renal Tubular Epithelial Cells

**FIGURE 2:**
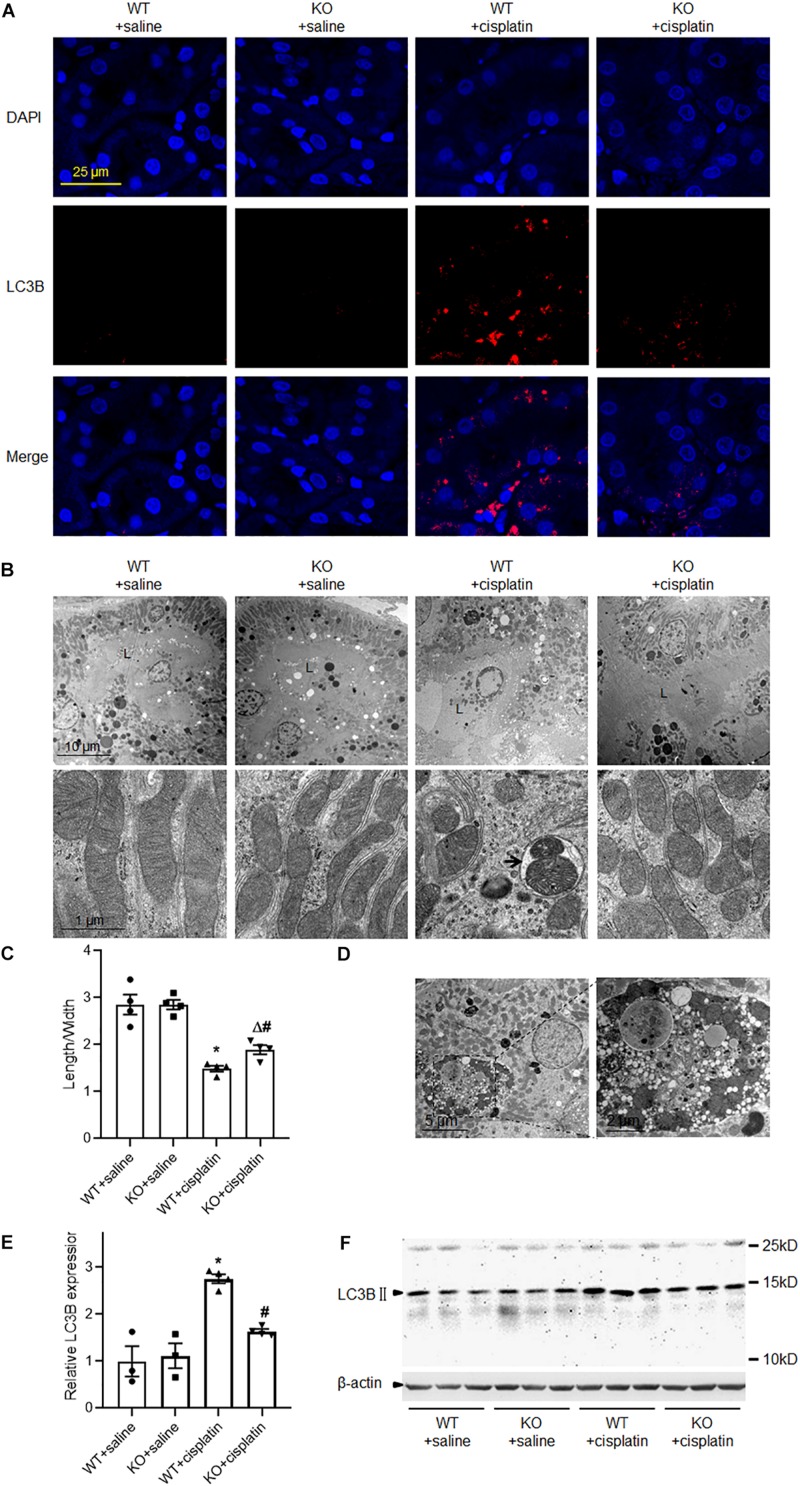
PINK1 deficiency prevented cisplatin-induced mitophagy. **(A)** Representative images of LC3B immunostaining. **(B)** Representative transmission electron microscope images of renal proximal tubular cells and mitochondria. L indicated the proximal tubular lumen. The arrow indicated a representative mitophagosome. **(C)** Quantitative analysis of the length/width of mitochondria in proximal tubular cells. **(D)** Transmission electron microscope images of large vacuoles containing numerous autophagosomes and mitophagosomes in cisplatin treated WT rats. **(E)** Quantitative analysis of the western blotting of LC3BII. **(F)** Representative western blot bands of LC3BII in renal cortex. *N* = 3 in WT + saline group and KO + saline group, *N* = 4 in WT + cisplatin group and KO + cisplatin group. ^∗^*P* < 0.05, WT + cisplatin vs. WT + saline; ^#^*P* < 0.05, KO + cisplatin vs. WT + cisplatin; ^Δ^*P* < 0.05, KO + cisplatin vs. KO + saline. Data are mean ± SE.

To address the effect of PINK1 deficiency on mitophagy during cisplatin treatment, we checked the expression of LC3B by western blot and immunofluorescence. As expected, cisplatin treatment significantly increased the expression of LC3B in renal proximal tubular epithelial cells and PINK1 deficiency blocked the up-regulation of LC3B (Figures 2A,E,F). We further verified cisplatin-induced autophagy by electron microscopy. As shown in Figure 2B, there were many autophagic vacuoles or vesicles in proximal tubular epithelial cells of WT rats after cisplatin treatment. Moreover, we found large vacuoles containing numerous autophagosomes and mitophagosomes in WT + cisplatin group (Figure 2D). Besides, after cisplatin treatment, many small and round mitochondria were observed and the ratios of mitochondrial lengths to widths were significantly decreased in proximal tubular cells of WT rats, indicating mitochondrial fragmentation (Figures 2B,C). Notably, all of these changes seemed to be attenuated by PINK1 deficiency.

### The Expression of PINK1 and Parkin During Cisplatin Treatment

**FIGURE 3:**
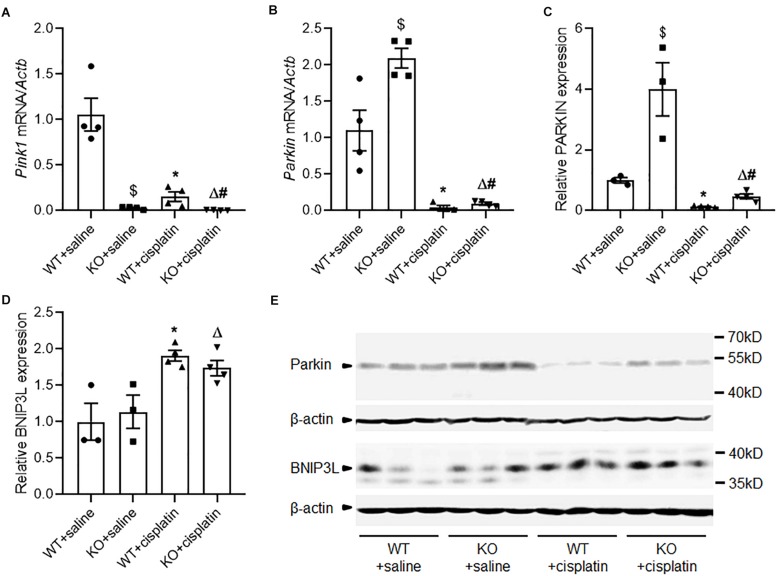
Altered expression of PINK1, Parkin and BNIP3L during cisplatin treatment in PINK1 WT and KO rats. **(A)** Real-time quantitative PCR analysis of *Pink1* mRNA expression in renal cortex. **(B)** Real-time quantitative PCR analysis of *Parkin* mRNA expression in renal cortex. **(C)** Quantitative analysis of the western blotting of Parkin. **(D)** Quantitative analysis of the western blotting of BNIP3L. **(E)** Representative western blot bands of Parkin and BNIP3L in renal cortex. *N* = 3 in WT + saline group and KO + saline group, *N* = 4 in WT + cisplatin group and KO + cisplatin group. ^∗^*P* < 0.05, WT + cisplatin vs. WT + saline; ^#^*P* < 0.05, KO + cisplatin vs. WT + cisplatin; ^Δ^*P* < 0.05, KO + cisplatin vs. KO + saline; ^$^*P* < 0.05, KO + saline vs. WT + saline. Data are mean ± SE.

Real-time PCR detected very little *Pink1* mRNA in PINK1 KO rats. This indicated that PINK1 KO was effective. In WT rats, cisplatin treatment significantly decreased the expression of *Pink1* (Figure 3A). However, we were unable to confirm the protein expression of PINK1 due to lack of specific antibodies against PINK1 protein in rats (Dave et al., 2014). For Parkin, cisplatin treatment decreased the mRNA and protein expression significantly but PINK1 KO increased the expression of Parkin both under cisplatin treatment conditions and unchallenged conditions (Figures 3B,C,E). The down-regulation of PINK1 and Parkin indicated that PINK1/Parkin pathway might not take the major responsibility for the active of mitophagy in cisplatin-induced kidney injury. BNIP3/BNIP3L pathway is another main mechanism of mitophagy (Ney, 2015). We checked the expression of BNIP3L by western blot. The results showed that cisplatin treatment distinctly increased the renal expression of BNIP3L, but there was no difference between PINK1 KO rats and WT rats (Figures 3D,E).

### The Effect of PINK1 Deficiency on Mitochondrial Dynamics During Cisplatin Treatment

**FIGURE 4:**
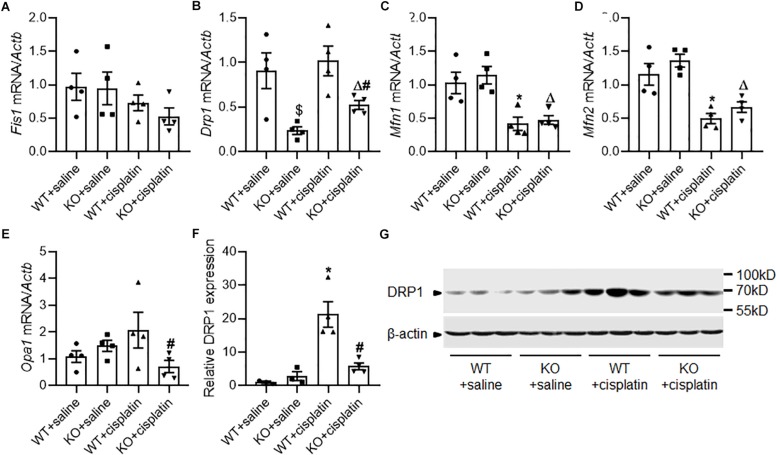
The effect of PINK1 deficiency on mitochondrial dynamics related proteins during cisplatin treatment. **(A–E)** Real-time quantitative PCR analysis of *Fis1*, *Drp1*, *Mfn1*, *Mfn2*, and *Opa1* mRNA expression in renal cortex. **(F)** Quantitative analysis of the western blotting of DRP1. **(G)** Representative western blot bands of DRP1 in renal cortex. *N* = 3 in WT + saline group and KO + saline group, *N* = 4 in WT + cisplatin group and KO + cisplatin group. ^∗^*P* < 0.05, WT + cisplatin vs. WT + saline; ^#^*P* < 0.05, KO + cisplatin vs. WT + cisplatin; ^Δ^*P* < 0.05, KO + cisplatin vs. KO + saline; ^$^*P* < 0.05, KO + saline vs. WT + saline. Data are mean ± SE.

Results from electron microscope showed mitochondrial fragmentation after cisplatin treatment indicated the change of mitochondrial dynamics from fusion to fission. So, we checked the mRNA expression of mitochondrial dynamics related genes including *Fis1*, *Drp1*, *Opa1*, *Mfn1*, and *Mfn2* by realtime PCR (Figures 4A–E). The results showed that mitochondrial fusion related genes *Mfn1* and *Mfn2* decreased significantly after cisplatin treatment both in WT and KO rats. However, *Drp1* which is critical for mitochondrial fission was significantly down-regulated in PINK1 KO rats both under cisplatin treatment conditions and unchallenged conditions. Subsequently, we double checked the protein expression of DRP1 by western blots. As shown in Figures 4F,G, cisplatin treatment induced an evident increase of DRP1 in renal cortex of WT rats, but it was largely attenuated in PINK1 KO rats, suggesting a key role of PINK1/Parkin in DRP1-mediated mitochondrial fission during cisplatin treatment.

### PINK1 Deficiency Attenuated Cisplatin-Induced Renal Tubular Cell Apoptosis

**FIGURE 5:**
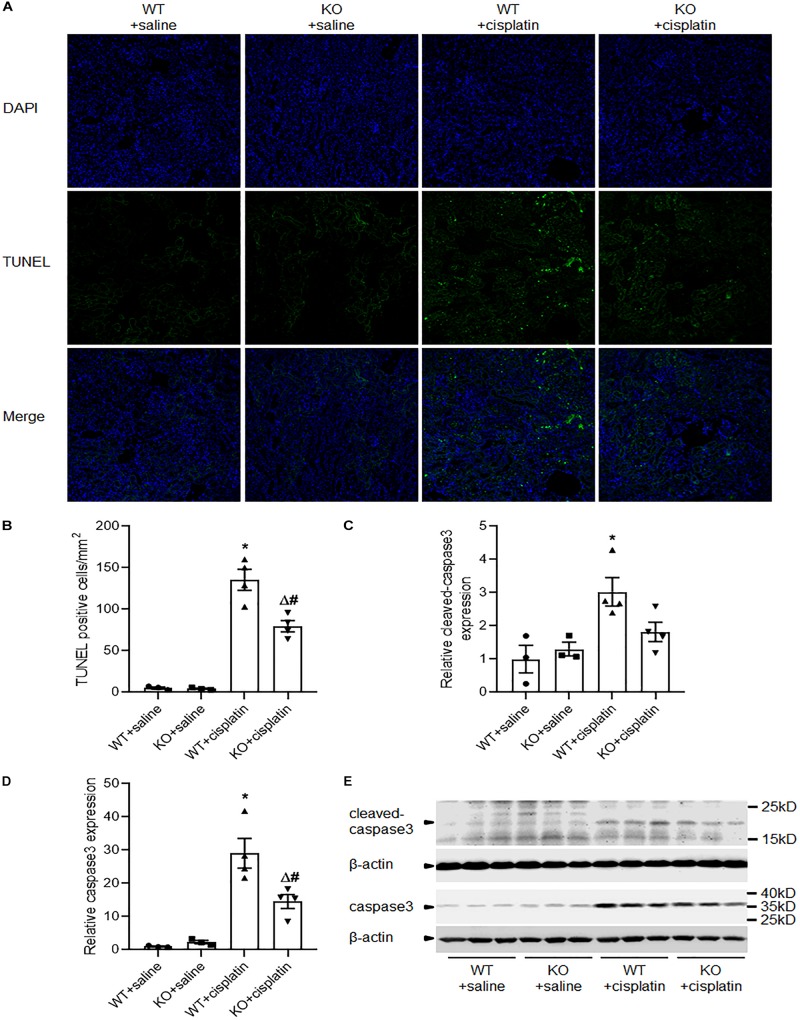
PINK1 deficiency attenuated cisplatin-induced renal tubular cell apoptosis. **(A)** Presented are the representative photographs of TUNEL staining of kidney tissues in PINK1 WT and KO rats after cisplatin treatment. **(B)** Quantitative analysis of TUNEL-positive cells among the various groups. **(C,D)** Quantitative analysis of the western blotting of cleaved caspase3 and caspase3. **(E)** Representative western blot bands of cleaved caspase3 and caspase3. *N* = 3 in WT + saline group and KO + saline group, *N* = 4 in WT + cisplatin group and KO + cisplatin group. ^∗^*P* < 0.05, WT + cisplatin vs. WT + saline; ^#^*P* < 0.05, KO + cisplatin vs. WT + cisplatin; ^Δ^*P* < 0.05, KO + cisplatin vs. KO + saline; ^$^*P* < 0.05, KO + saline vs. WT + saline. Data are mean ± SE.

Apoptosis and autophagy are highly coordinated mechanisms in response to various intrinsic and/or extrinsic stresses to maintain cellular homeostasis and it is well known that apoptosis contributes to cisplatin nephrotoxicity. We evaluated renal tubular cell apoptosis by TUNEL assay (Figures 5A,B). The results revealed that there were significantly less TUNEL-positive tubular cells in KO rats than WT rats after cisplatin treatment. Next, we checked the expression of caspase3 and cleaved caspase3 by western blots. Consistent with TUNEL assay, immunoblot analysis also showed a lower induction of caspase3 and cleaved caspase3 in renal cortex after cisplatin treatment in PINK1 KO rats (Figures 5C–E).

## Disscusion

Many previous studies have clearly demonstrated mitochondrial damage and dysfunction as a key pathophysiological component in cisplatin nephrotoxicity. The pathological changes of mitochondria in cisplatin nephrotoxicity are mainly triggered by DNA damage response, disruption of mitochondrial dynamics, pro-apoptotic protein attack and oxidative stress (Yang et al., 2014). There is evidence that mitochondrial DNA and other mitochondrial targets are perhaps more important than nuclear DNA damage in mediating cisplatin-induced cell death and the sensitivity of cells to cisplatin seems to correlate well with mitochondrial density and mitochondrial membrane potential (Peres and da Cunha, 2013). Mitochondrial damage and dysfunction would then result in abnormal energy metabolism, oxidative stress, inflammation, and cell death, inducing a rapid deterioration in renal function (Manohar and Leung, 2018). Therefore, maintenance of a cohort of healthy mitochondria is crucial for the homeostasis and viability of renal tubular cells in cisplatin-induced kidney injury.

PINK1/Parkin-mediated mitophagy pathway is one of the best studied mechanisms for mitophagy in mammalian cells (Eiyama and Okamoto, 2015). Recently, Wang et al. (2018) demonstrated that renal PINK1 and Parkin were increased in cisplatin treated mice and indicated that PINK1/Parkin-mediated mitophagy had a protective role against kidney injury. Besides, there is evidence that PINK1-parkin pathway of mitophagy protects against contrast-induced acute kidney injury via decreasing mitochondrial ROS and NLRP3 inflammasome activation (Lin et al., 2019). However, in our present study, we found renal PINK1 and Parkin were significantly down-regulated in cisplatin treated rats. This is surprising but not impossible. In streptozotocin-induced diabetic mice and db/db mice, Li et al. and Sun et al. found mitochondrial abnormalities occurred in the kidney accompanied by reduced PINK1 and Parkin expression, while BNIP3 and LC3B were significantly increased in the renal tubular cells (Huang et al., 2016; Li et al., 2017; Xiao et al., 2017). There are many possible reasons for the different pattern of PINK1 expression and role between cisplatin treated mice and rats. First, autophagy may be a double-edged sword. Indeed, stimulation of autophagy has been described to both improve and exacerbate ischemia-reperfusion injury in the kidney and a hypothesis has been put forward that autophagy can switch roles depending on the severity of the kidney injury and the phase of the injury process (Decuypere et al., 2015). Moderate level of autophagy may improve kidney injury and excessive autophagy may exacerbate kidney injury (Decuypere et al., 2015). The different cisplatin doses and differential accumulation of cisplatin in mitochondria between rats and mice in the absence of PINK1 in mitochondria may result in different mitochondria injury severity between rats and mice. In fact, we found a number of autophagic vacuoles or vesicles as well as large vacuoles containing numerous autophagosomes and mitophagosomes in cisplatin treated WT rats, indicating excessive autophagy. And the excessive mitochondria damage and autophagy might have negative feedback inhibition effect on the expression of PINK1 and Parkin. Second, most of evidences that autophagy exacerbated renal ischemia-reperfusion injury were found in rats models (Decuypere et al., 2015). Considered the difference that we used PINK1 KO rats and Wang et al. (2018) used PINK1 KO mice, it may also indicate species difference between mice and rats. Besides, aging are associated with dysfunctional autophagy, which may reduce the threshold for detrimental autophagy (Filfan et al., 2017). Furosemide is frequently used in patients with acute kidney injury to promote diuresis. Interestingly, furosemide can induce autophagosomes in the thick ascending loop and it has been reported that the detrimental effect of furosemide only occurs in adult rats but partially protective in young rats during renal ischemia-reperfusion injury (Fleck and Heller, 1993). The age of rats (24–28 week old) may also contribute to the protective effects of PINK1 deficiency that observed in present study. In addition, the different gene targeting strategies between KO rats and KO mice that result in different products of transcription from *Pink1* locus could also be a reason.

In present study, electron-microscopy and LC3B expression indicated mitophagy was activated in cisplatin-induced acute kidney injury. However, real-time PCR and western blots showed a down-regulation of PINK1 and Parkin by cisplatin treatment, suggesting other mechanisms of mitophagy. There are substantial evidences that BNIP3 and BNIP3L can promote mitochondrial depolarization and mitophagy (Ney, 2015). Ishihara et al. reported that BNIP3 might regulate mitophagy in the proximal tubules of ischemia-reperfusion injury rats (Ishihara et al., 2013). In this study, we checked the expression of BNIP3L and found that cisplatin significantly up-regulated the expression of BINP3L, indicating BNIP3/BNIP3L pathway may play a major role for the elevated mitophagy in cisplatin treated rats.

Mitochondria are highly dynamic organelles that shift between fragmented and tubule like morphologies by the control of mitochondria fission and fusion. A number of biochemical and genetic studies link the PINK1/Parkin pathway to mitochondrial fission and fusion (Voigt et al., 2016). It has been demonstrated that normally tubule like mitochondria in the proximal tubules rapidly fragment in response to insults such as cisplatin and ischemia-reperfusion injury (Hall et al., 2013). Emerging evidences suggest that the mitochondrial fragmentation caused by changes of mitochondria fusion and fission balance has a pathogenic role in acute kidney injury. In line with those studies, electron-microscopy showed increased mitochondrial fragmentation after cisplatin treatment in WT rats while PINK1 deficiency seemed to decrease mitochondrial fragmentation in present study. The molecular machinery governing mitochondrial dynamics is determined by fission proteins, such as DRP1 and FIS1, and fusion proteins, such as MFN1, MFN2, and OPA1 (Meyer et al., 2017). Brooks et al. demonstrated in both cell and mouse models that DRP1 is rapidly recruited to fragmented mitochondria in the proximal tubule and inhibition of DRP1 was protective against mitochondrial fragmentation and kidney damage (Brooks et al., 2009). In present study, PINK1 deficiency remarkably restrained cisplatin-induced DRP1 up-regulation, indicating the regulation of DRP1 by PINK1/Parkin pathway might play a key role in cisplatin-induced acute kidney injury. A number of studies demonstrated that the PINK1/Parkin pathway is pro-fusion as overexpression of PINK1 leads to elongated mitochondria while knockdown of PINK1 leads to fragmented mitochondria (Dagda et al., 2009; Lutz et al., 2009). Accordantly, in present study cisplatin-induced mitochondrial fragmentation was accompanied by a down-regulation of PINK1 and Parkin. Interesting, we found PINK1 deficiency induced a compensatory increase of Parkin expression both under cisplatin treatment conditions and unchallenged conditions. And this compensatory increase of Parkin might account for the decrease of mitochondrial fragmentation and DRP1 expression in PINK1 KO rats during cisplatin treatment. In agree with this, Buhlman et al. (2014) demonstrated that Parkin-mediated mitochondrial fission depends on DRP1 and at least in part independently of PINK1. Additionally, mitochondria need to be fragmented before mitophagy to occur. Zhao et al. (2017a) demonstrated that DRP1 dependent mitochondrial fission was required for mitophagy in cisplatin treated tubular epithelial cells. Consistently, Li et al. (2018) showed that renal ischemia-reperfusion induced mitophagy also depended on DRP1-mediated mitochondrial fragmentation. Besides, *Drp1* mRNA expression was unchanged but DRP1 protein level was significantly up-regulated by cisplatin treatment in WT rats, indicating a main role of post-transcriptional regulation. However, PINK1 deficiency significantly inhibited *Drp1* mRNA expression both under cisplatin treatment conditions and unchallenged conditions, indicating a main role of transcriptional regulation. And the different pattern of *Drp1* mRNA expression and DRP1 protein level might suggest that *Drp1* mRNA expression was ample under normal conditions and the effect of PINK1 deficiency on *Drp1* transcriptional regulation was covered up. Under cisplatin treatment conditions, *Drp1* mRNA were extensively transformed into DRP1 proteins and then the effect of PINK1 deficiency on *Drp1* transcriptional regulation appeared. Interesting, it has been reported that Parkin could also act as a transcription factor and modulate various genes (Alves da Costa et al., 2018). Thus, there was high possibility that the effect of PINK1 deficiency on *Drp1* transcriptional regulation was related to the compensatory increase of Parkin expression. Taken together, it was probable that PINK1 deficiency caused a compensatory increase of Parkin and affected DRP1 dependent mitochondrial fission to protect against mitochondrial fragmentation, mitophagy and overall kidney damage.

It is well known that apoptosis contributes to cisplatin nephrotoxicity and some studies suggest that apoptosis and autophagy are co-ordinated processes in the pathogenesis of acute kidney injury (Zhou et al., 2018). The dual role of autophagy or mitophagy under stimulating factor may depend on the balance between pro-survival and pro-death processes. Generally, when cell is exposed to modest stress and just produce a few damaged mitochondria, the cell can easily remove those abnormal mitochondria via mitophagy. However, if plenty of mitochondria are damaged leading to insufficient scavenging capacity, the cell is beyond rescue and apoptosis will become the dominant pathway (Kubli and Gustafsson, 2012). Therefore, it is not surprising that both apoptosis and mitophagy/autophagy were attenuated in PINK1 KO rats in present study and this may also indicate excessive mitophagy/autophagy during cisplatin-induced acute kidney injury. Besides, mitochondrial dynamics are integrated with mitophagy and cell death. Studies have shown that excessive DRP1-mediated mitochondrial fission contributes to apoptosis (Frank et al., 2001). It has been found that DRP1 and Bax are co-localized on the mitochondrial membrane at the onset of apoptosis and inhibition of DRP1 protected against irradiation and senecionine induced apoptosis (Wang et al., 2015; Zhang et al., 2016; Yang et al., 2017). However, there is also evidence that DRP1-mediated fission is not necessary for apoptosis process but it increases sensitivity to apoptotic signals possibly by enhancing cytochrome c release (Sugioka et al., 2004; Parone et al., 2006). Therefore, the reduced apoptotic level in PINK1 KO rats may partly due to the inhibition of DRP1 and mitochondrial fission during cisplatin-induced acute kidney injury.

**FIGURE 6:**
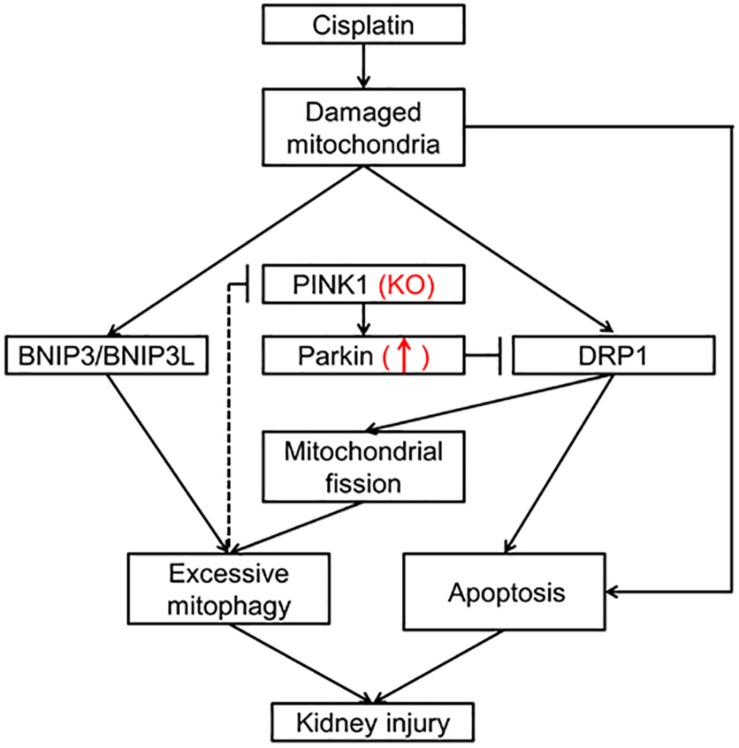
Schematic representation of possible mechanism related to mitophagy in cisplatin-induced kidney injury in PINK1 KO rats. Cisplatin causes the mitochondrial damage of renal tubular epithelial cells, which induces BNIP3/BNIP3L-mediated mitophagy, DRP1-mediated mitochondrial fission and apoptosis leading to kidney injury. And the excessive mitophagy may have negative feedback inhibition effect on the expression of PINK1 and Parkin and result in the down-regulation of PINK1 and Parkin in WT rats. In PINK1 KO rats, PINK1 deficiency causes a compensatory increase of Parkin and significantly inhibits the up-regulation of DRP1, which regulates mitochondrial fission and apoptosis, and therefore to inhibit the excessive mitophagy and overall kidney damage.

In summary, our results showed that renal mitophagy was activated in cisplatin treated rats and PINK1 deficiency ameliorated cisplatin-induced acute kidney injury accompanied by a decrease of mitophagy. Mechanistically, cisplatin treatment decreased the renal expression of PINK1 and Parkin while increased the expression of BNIP3L, indicating PINK1/Parkin mediated mitophagy might not play a major role in cisplatin nephrotoxicity rat models. Nevertheless, PINK1 deficiency caused a compensatory increase of Parkin and significantly inhibited cisplatin-induced up-regulation of DRP1, suggesting a renal protection mechanism by regulating DRP1 dependent mitochondrial fission and therefore to inhibit the excessive mitophagy and overall kidney damage (Figure 6).

## Data Availability Statement

The raw data supporting the conclusions of this manuscript will be made available by the authors, without undue reservation, to any qualified researcher.

## Ethics Statement

The animal study was reviewed and approved by Institutional Animal Care and Use Committee of the Institute of Laboratory Animal Science of Peking Union Medical College.

## Author Contributions

LZho performed the research, analyzed the data and wrote the manuscript. YZ, XY, and XL performed the animal experiments. YH contributed to the histopathological examination. LZha and WL performed the Western blotting. TZ and XS performed other experiments. CQ designed and funded the research. All authors have read and agreed with the manuscript.

## Conflict of Interest

The authors declare that the research was conducted in the absence of any commercial or financial relationships that could be construed as a potential conflict of interest.

## Abbreviations

BNIP3: BCL2/adenovirus E1B 19 kDa protein-interacting protein 3
BNIP3L: Bcl-2/adenovirus E1B 19 kDa-interacting protein 3-like proteinalso known as Nix
BUN: blood urea nitrogen
DAPI: 4′,6-diamidino-2-phenylindole
Drp1: dynamin related protein 1
Fis1: mitochondrial fission 1 protein
KO: knockout
LC3B: microtubule-associated protein light chain 3B
Mfn1: Mitofusin 1
Mfn2: Mitofusin 2
Opa1: optic atrophy 1
PAS: periodic acid schiff reaction
PINK1: PTEN induced putative kinase 1
TUNEL: TdT-mediated dUTP Nick-End Labeling
WT: wild-type.
