# Supplementary figures and images for: PINK1 Deficiency Ameliorates Cisplatin-Induced Acute Kidney Injury in Rats
Source: Front Physiol. 2019 Sep 25;10:1225. doi: 10.3389/fphys.2019.01225 (PMC6773839; doi:10.3389/fphys.2019.01225)

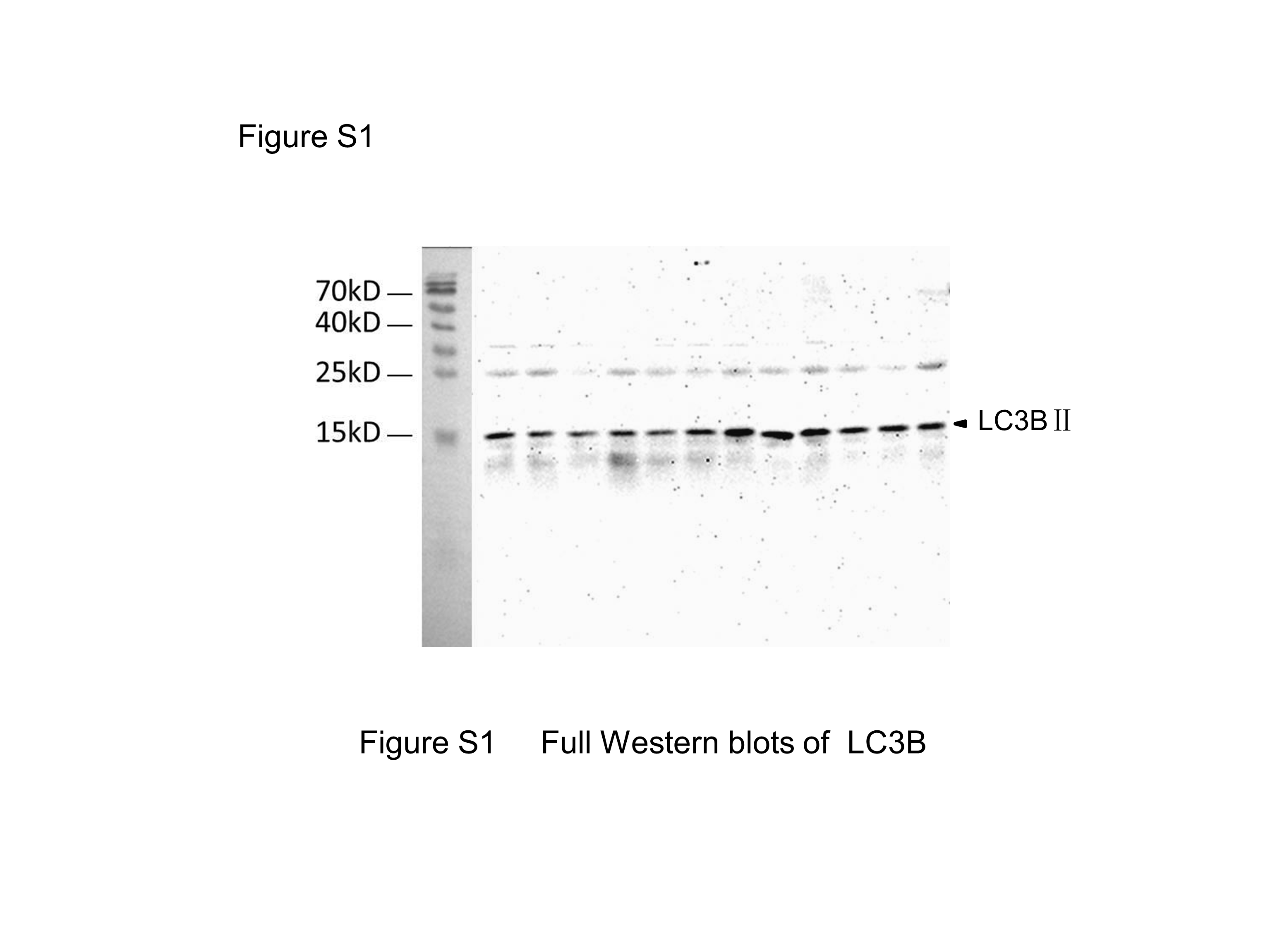

Supplement: Supplementary file 1 [file Image_1.TIF]

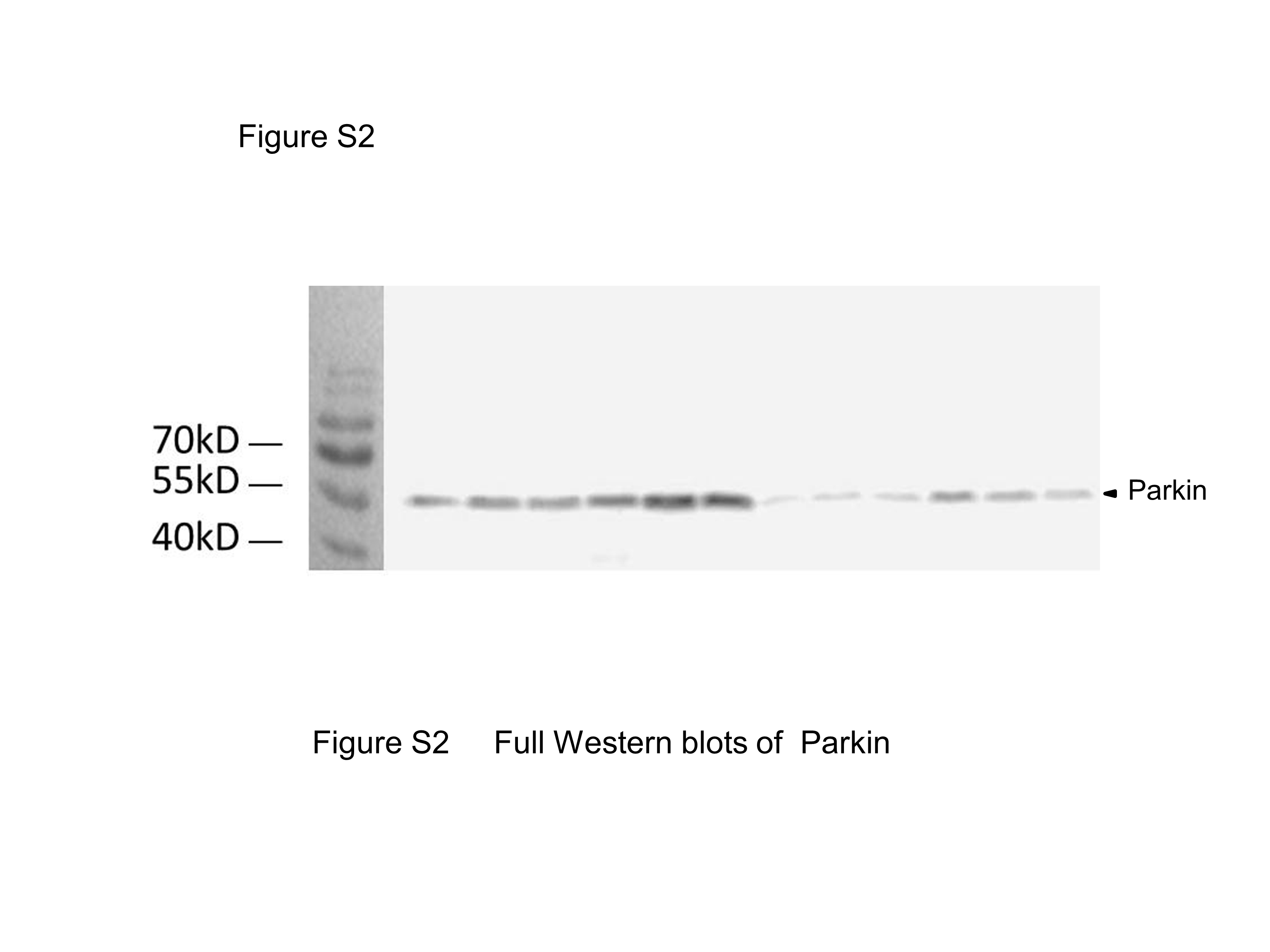

Supplement: Supplementary file 2 [file Image_2.TIF]

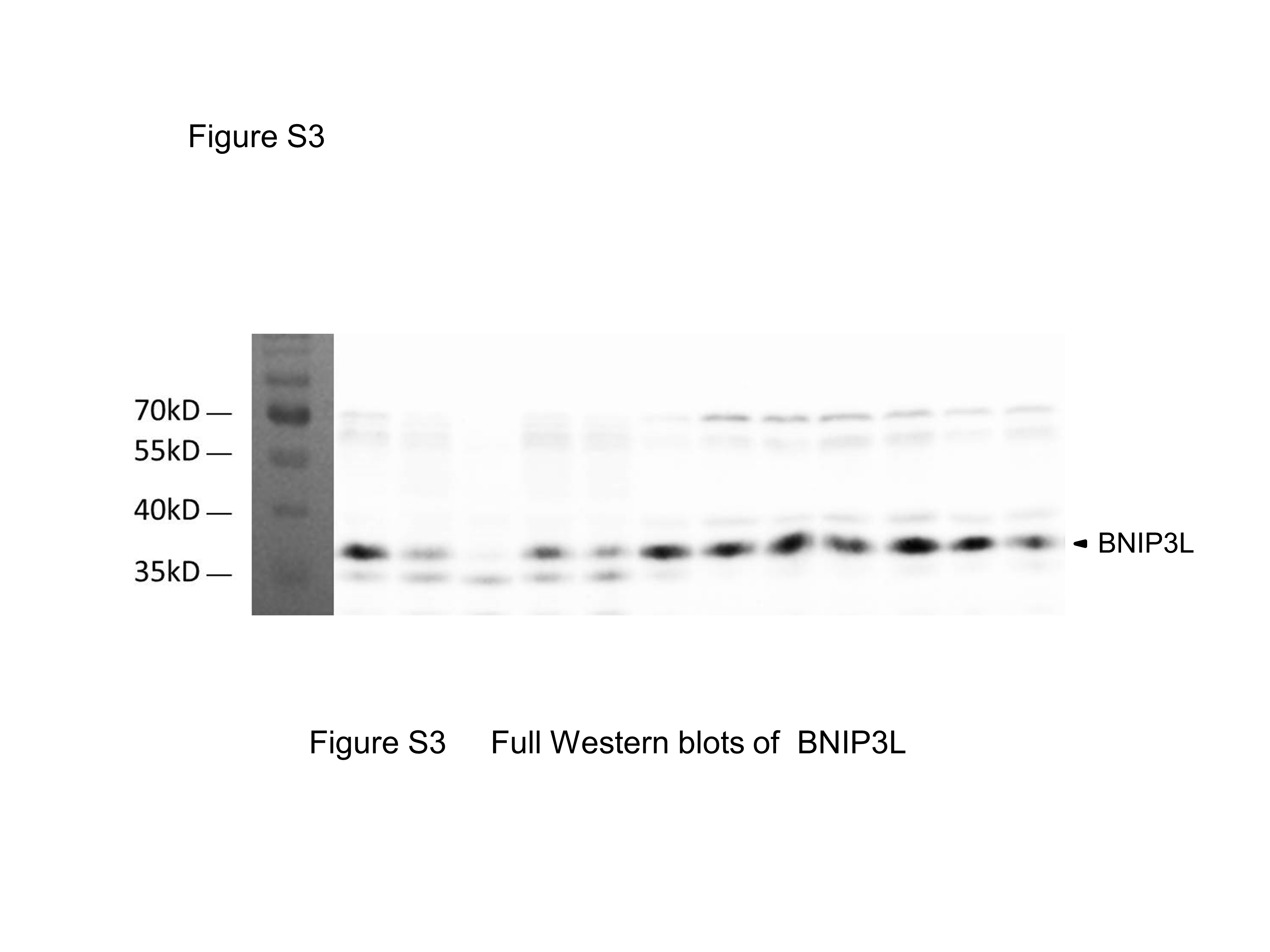

Supplement: Supplementary file 3 [file Image_3.TIF]

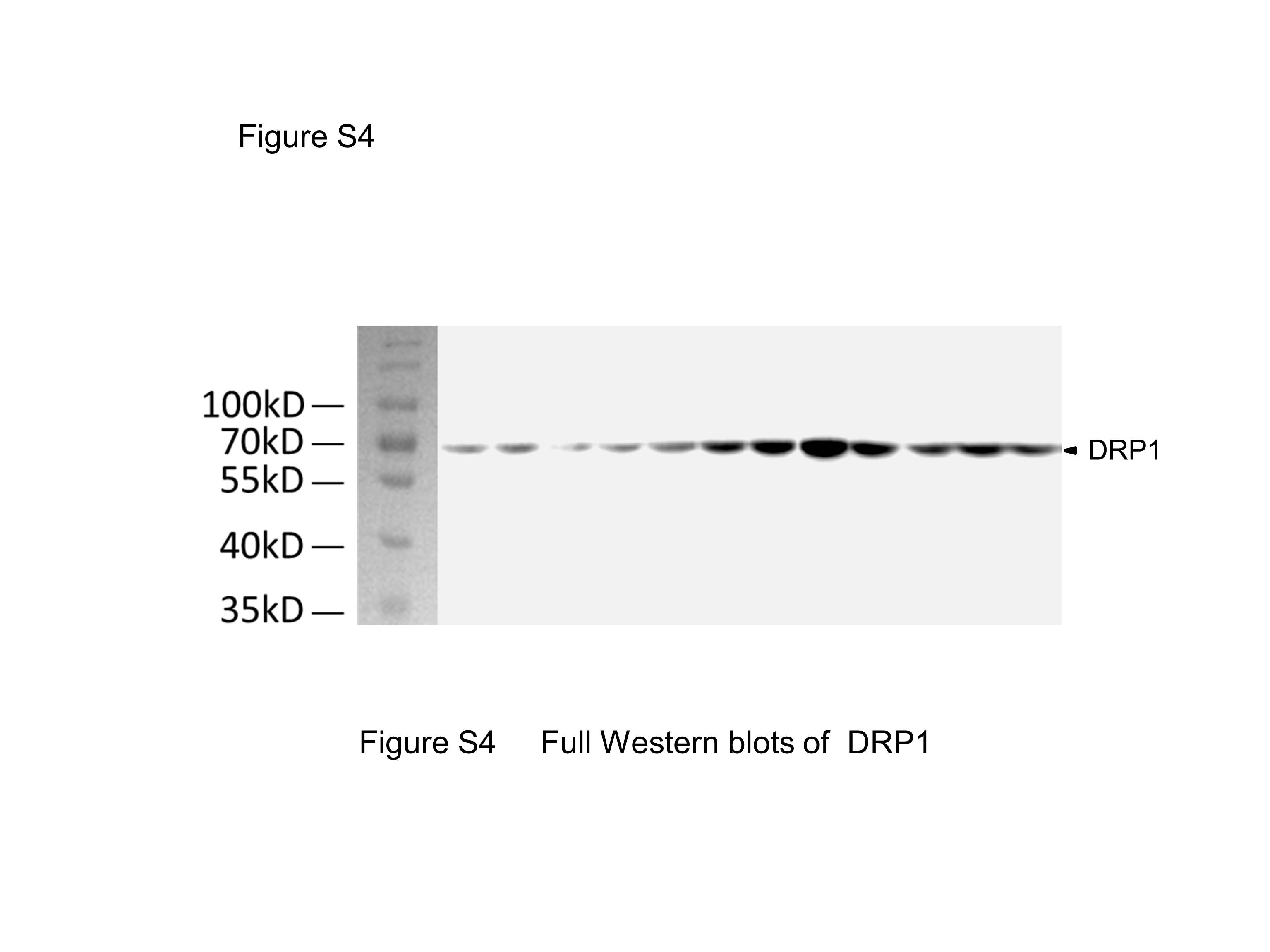

Supplement: Supplementary file 4 [file Image_4.TIF]

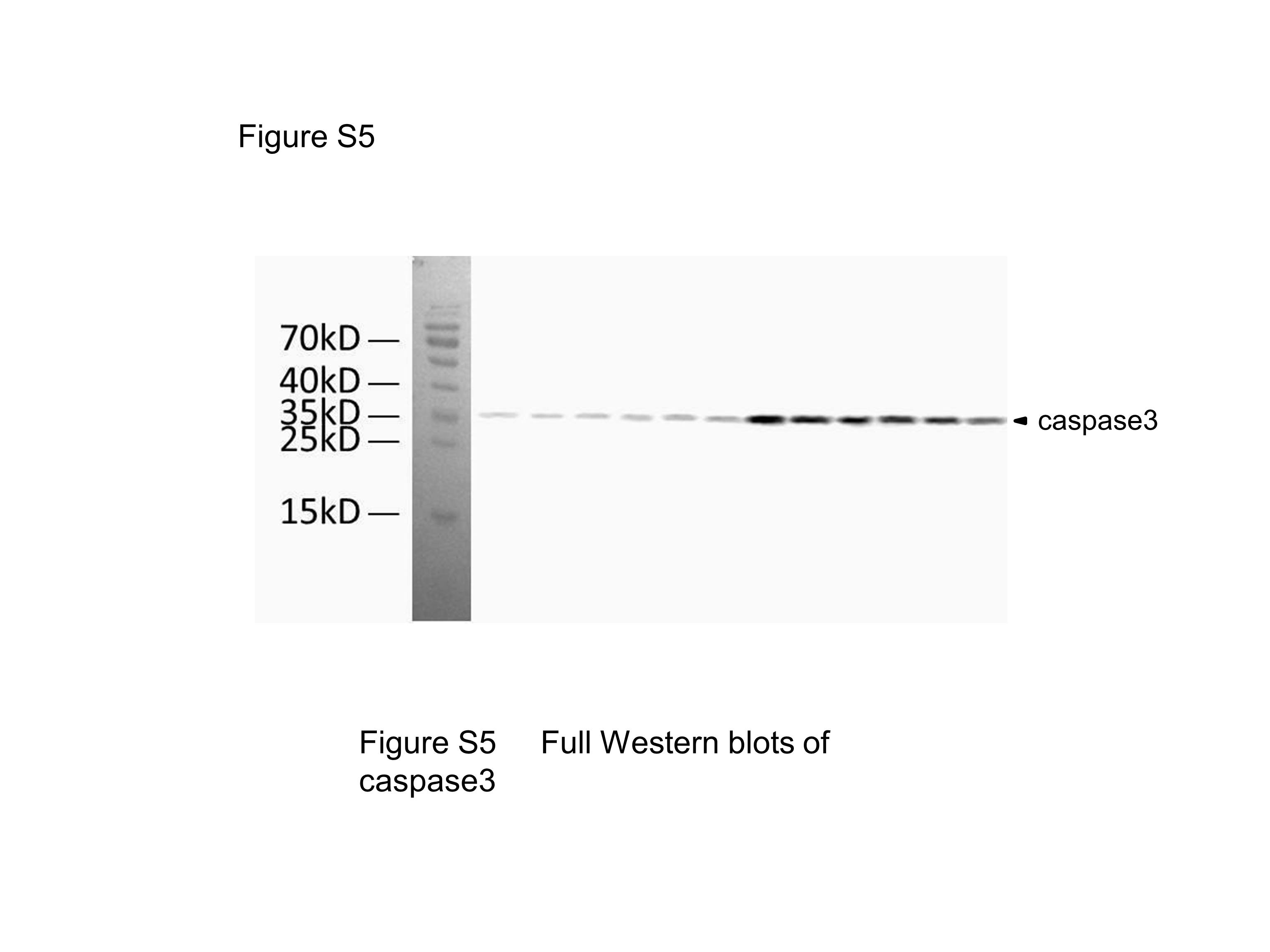

Supplement: Supplementary file 5 [file Image_5.TIF]

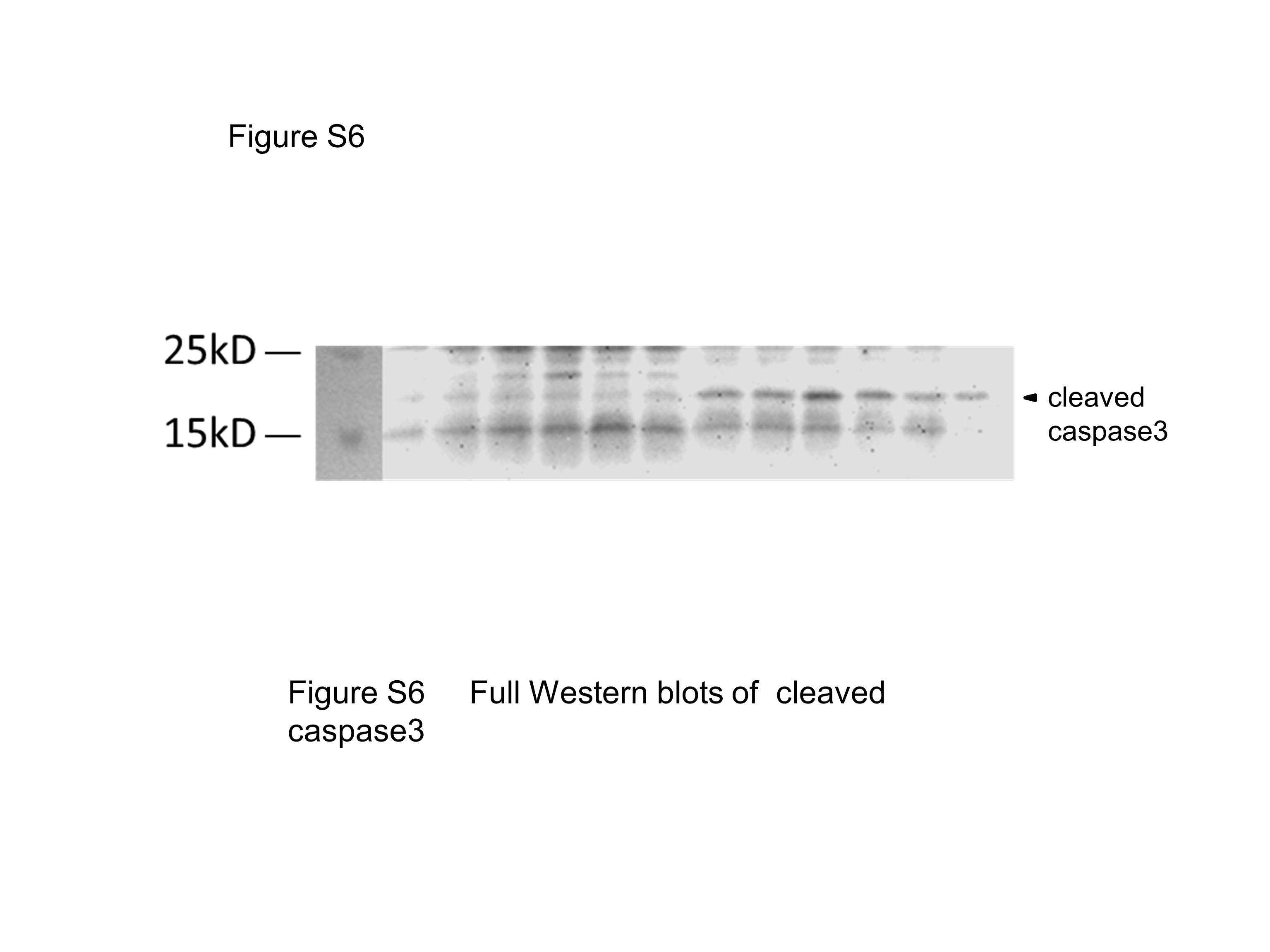

Supplement: Supplementary file 6 [file Image_6.TIF]
